# Synonymous Codon Usage Analysis of Three Narcissus Potyviruses

**DOI:** 10.3390/v14050846

**Published:** 2022-04-19

**Authors:** Zhen He, Shiwen Ding, Jiyuan Guo, Lang Qin, Xiaowei Xu

**Affiliations:** 1 School of Horticulture and Plant Protection, Yangzhou University, Yangzhou 225009, China; dinshiwen1012@163.com (S.D.); qinlanguihi@163.com (L.Q.); xxw6660620@163.com (X.X.); 2 Joint International Research Laboratory of Agriculture and Agri-Product Safety of Ministry of Education of China, Yangzhou University, Yangzhou 225009, China; 3 Department of Resources and Environment, Moutai Institute, Zunyi 564507, China; guojiyuan@163.com

**Keywords:** codon usage bias, potyviruses, narcissus, natural selection

## Abstract

Narcissus degeneration virus (NDV), narcissus late season yellows virus (NLSYV) and narcissus yellow stripe virus (NYSV), which belong to the genus *Potyvirus* of the family *Potyviridae*, cause significant losses in the ornamental value and quality of narcissus. Several previous studies have explored the genetic diversity and evolution rate of narcissus viruses, but the analysis of the synonymous codons of the narcissus viruses is still unclear. Herein, the coat protein (CP) of three viruses is used to analyze the viruses’ phylogeny and codon usage pattern. Phylogenetic analysis showed that NYSV, NDV and NLSYV isolates were divided into five, three and five clusters, respectively, and these clusters seemed to reflect the geographic distribution. The effective number of codon (ENC) values indicated a weak codon usage bias in the CP coding region of the three narcissus viruses. ENC-plot and neutrality analysis showed that the codon usage bias of the three narcissus viruses is all mainly influenced by natural selection compared with the mutation pressure. The three narcissus viruses shared the same best optimal codon (CCA) and the synonymous codon prefers to use codons ending with A/U, compared to C/G. Our study shows the codon analysis of different viruses on the same host for the first time, which indicates the importance of the evolutionary-based design to control these viruses.

## 1. Introduction

Narcissus (*Narcissus tazetta var. chinensis*) is an economically important vegetatively propagated monocotyledon plant, which is susceptible to an accumulation of viruses. Presently, more than twenty viruses belonging to the genera *Potyvirus*, *Carlavirus*, *Macluravirus*, *Potexvirus* and *Nepovirus* were identified from narcissus [1,2,3,4]. Narcissus degeneration virus (NDV), narcissus late season yellows virus (NLSYV) and narcissus yellow stripe virus (NYSV), belonging to the genus *Potyvirus* of the family *Potyviridae*, are the most frequently seen viruses infecting narcissus [3,5,6,7]. NDV and NLSYV were first reported in plants of *N. tazetta* and *N. pseudonarcissus* by Brunt et al. [8]. NYSV infection in plants of *N. spp.* was reported from Australia, China, the Eurasian region, Japan and India [1,3,6,7,9]. NDV, NLSYV and NYSV contain positive-sense single-stranded RNA molecules with a genome size of approximately 10 kb, encapsidated by an approximately 750-nm-long flexuous filamentous virion [3,6,7,10]. In nature, the infection of NDV, NLSYV and NYSV are mostly limited to narcissus [1].

It is widely known that cordons encoding the same amino acid are considered synonymous codons. Codon usage bias refers to the phenomenon that various organisms prefer to use synonymous triplet codons (that is, codons that encode the same amino acid) [11]. There are big differences in gene codon usage in different species and different organisms [12]. In the absence of selection pressure and neutral mutations, several synonymous codons encoding the same amino acid should be used at the same frequency. In 1980, Grantham et al. proposed the genome hypothesis, which believed that codon bias is species-specific, that is, within the same species or between species with similar genetic relationships, they generally show similar codon usage patterns [13]. From prokaryotes to eukaryotes, the phenomenon of synonymous codon usage bias in the genome is widespread. This phenomenon is related to many factors, such as the base composition of gene sequence, natural selection effect, tRNA abundance, gene length, protein structure and function, protein hydrophobicity level and amino acid conservation, etc. [14,15,16,17,18,19].

However, the synonymous codon usage bias of the narcissus viruses is largely unclear. In this study, we have determined 26 coat protein (CP) sequences of three potyviruses (NYSV, NDV and NLSYV) isolated from narcissus in Jiangsu Province. We analyzed three viruses’ codon usage bias based on the coat protein coding region sequences and explored factors that might be related to this codon usage bias. Our study shows the codon analysis of different viruses on the same host for the first time and will provide a theoretical basis for controlling the spread of viruses.

## 2. Materials and Methods

### 2.1. Virus Isolates

**Figure 1:**
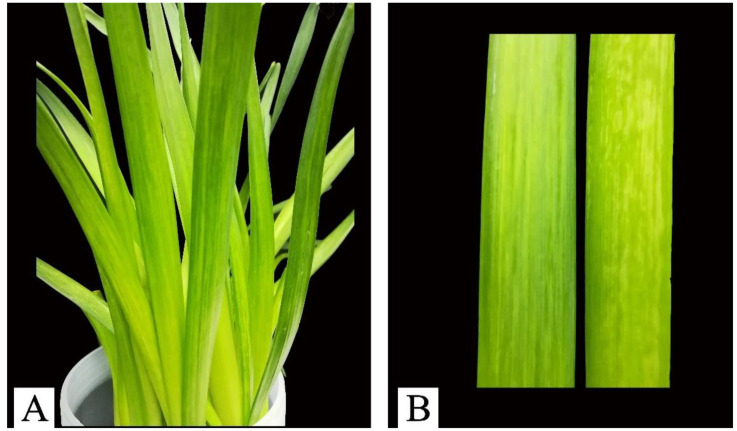
The Chinese narcissus plants are infected with potyviruses. (**A**) The potyviruses-infected narcissus plants in Yangzhou city of Jiangsu province, China; (**B**) Leaves of the Chinese narcissus plants showing mosaic and chlorotic stripes symptoms.

The Chinese narcissus plants showing mosaic and chlorotic stripes symptoms (Figure 1) were collected from the home garden in Yangzhou City of Jiangsu province in China during 2016–2017. The fresh leaves were stored at −80 °C until use.

### 2.2. Viral RNA and Sequencing

The viral RNAs were extracted from 100 mg Chinese narcissus leaves using TRIzol^™^ reagent (Invitrogen, Shanghai China). For cDNA synthesis and amplification of the *CP* gene, we used a Phanta^®^ Max Super-Fidelity DNA Polymerase RT-PCR kit (Vazyme, Nanjing, China) with primers POTYNIbNOT4P and Tu3T9M [1]. The RT-PCR products were separated by electrophoresis in agarose gels and purified using the FastPure^®^ Gel DNA Extraction Mini Kit (Vazyme, Nanjing, China). The resulting fragments were cloned into the pMD19-T vector (Takara, Dalian, China). The recombinant DNA was transformed into *Escherichia coli* DH5α. Twenty-six *CP* gene sequences of three potyviruses (4 isolates of NYSV, 16 isolates of NDV and 6 isolates of NLSYV, respectively) isolated from narcissus in Yangzhou were determined here. All available CP sequences of the three potyviruses were obtained from the GenBank database. Sequence data were assembled using BioEdit v7.0.9 (Borland, Scotts Valley, CA, USA).

### 2.3. Phylogenetic and Recombination Analysis

The phylogenetic of the multiple aligned sequences were inferred by the maximum likelihood method (ML) to analyze the evolutionary relationship implemented in MEGA v11 [20]. The branch support was evaluated by the bootstrap method (bootstrap = 1000) in the ML tree. For the ML tree, the best-fit model of nt substitutions for each dataset was determined using jModeltest v0.1.1 [21]. The calculated trees were displayed by TREEVIEW [22]. Sequence alignment in open reading frame (ORF) by software CLUSTAL X2 [23], and putative recombination sites in the aligned sequences were identified using the BOOTSCAN [24], GENECONV, MAXCHI [25], RDP [26], CHIMAERA [27], SISCAN [28] and 3SEQ [29] programs in the RDP4 software package. First, analysis of potential recombination events was completed using RDP4. The recombinant site was detected by RDP4 software and the possible parent sequence was determined. All sites were examined with an associated *p*-value of <1 × 10^−6^ (the most likely recombination sites).

### 2.4. Relative Synonymous Codon Usage (RSCU) Analysis

The RSCU value is an effective indicator to measure the degree of codon usage bias [30]. The RSCU value calculation formula is as follows:

$${\mathrm{RSCU}}_{\mathrm{ij}}=\frac{g_{\mathrm{ij}}}{\sum_{j}^{\mathrm{ni}}g_{\mathrm{ij}}}\times \mathrm{ni}$$

In the formula, g_ij_ is the j-th codon of the i-th amino acid actual observations and ni is the number of codons encoding amino acid i. When the theoretical value of RSCU is equal to the expected value, there is no codon bias. Therefore, when RSCU > 1.0 is the actual high-frequency codon; RSCU < 1.0 is the actual low frequency codon usage [31]. The RSCU values of NYSV, NDV and NLSYV CP sequences and narcissus genes were computed using MEGA v11 software [20].

### 2.5. Effective Number of Codons (ENC) Analysis

ENC was calculated using codon W1.4.2 software. The ENC values range is usually 20 (the extreme case of using only 1 codon per amino acid)–61 (all codons are used equally), which is commonly used to evaluate the codon preference analysis of a single gene. Generally, the lower the ENC value, the stronger the preference [32,33].

### 2.6. Principal Component Analysis (PCA)

PCA is a multivariate statistical method to explore codon usage bias and its influencing factors. PCA distributed all the target genes studied on the vector axis, and used the dimension reduction method to find the four vector axes that played a major role, namely four principal components: Axis1, Axis2, Axis3 and Axis4 [34,35]. Each strain was recognized as a 59-dimensional vector and then the RSCU value of each synonymous codon corresponds to each dimension. The three termination codons, UGG and AUG were excluded from the analysis. PCA was analyzed using Origin 9.1 (OriginLab, Northampton, MA, USA) [36].

### 2.7. ENC-Plot Analysis

The ENC-plot analysis is used to investigate the decisive factor of codon preference and the abscissa is the GC3s value while the ordinate is the ENC value. The distance of each point in the ENC-plot diagram from the expected curve can reflect that the reason for the formation of codon preference is a base mutation or natural selection [37]. If the codon preference formation of a gene is significantly affected by mutations, its ENC-GC3s points will be on the standard curve; if it is greatly affected by natural selection, it will be distributed further away from the standard curve. The ENC-plot formula is as follows:

$$\mathrm{ENC expected}=2+s+\left(\frac{29}{s^{2}+{\left(1-s\right)}^{2}}\right)$$

In the form, s represents the value of GC3s [32].

### 2.8. Neutrality Analysis

The neutral plot is used to compare the impact of mutation pressure and natural selection on codon usage patterns. The abscissa and ordinate are GC3 and GC12, respectively [38]. Mutations in synonymous codons generally exist in the third codon position, while those in the first or second basic group are mutations in non-synonymous codons. If the slope of the regression curve is close to one, the correlation between GC12 and GC3 is significant, indicating that the codon bias is mainly affected by the mutation. In contrast, the correlation between GC12 and GC3 is not obvious, the slope of the regression curve is close to 0 and the codon usage is mainly affected by selection [39].

### 2.9. Parity Rule 2 (PR2) Analysis

PR2 plot analysis was performed to determine the factors affecting genomic codon bias. Here, the scatter plot is plotted with G3/(G3 + C3) as abscissa and A3/(A3 + U3) as ordinate. The center of the plot is where A = U and G = C (PR2), showing no deviation between gene mutation and natural selection in codon usage [40,41].

## 3. Results

### 3.1. Nucleotide Sequences

In total, three potyviruses (NYSV, NDV and NLSYV) were detected from Chinese narcissus plants. Twenty-six CP nucleotide sequences of these three potyviruses were determined here and submitted to the GenBank database with the accession codes shown in Table S1. The CP coding region sequences of NYSV, NDV and NLSYV were 825, 786 and 825 nucleotides (nt) in length, respectively. 

### 3.2. Recombination and Phylogenetic Analysis

**Figure 2:**
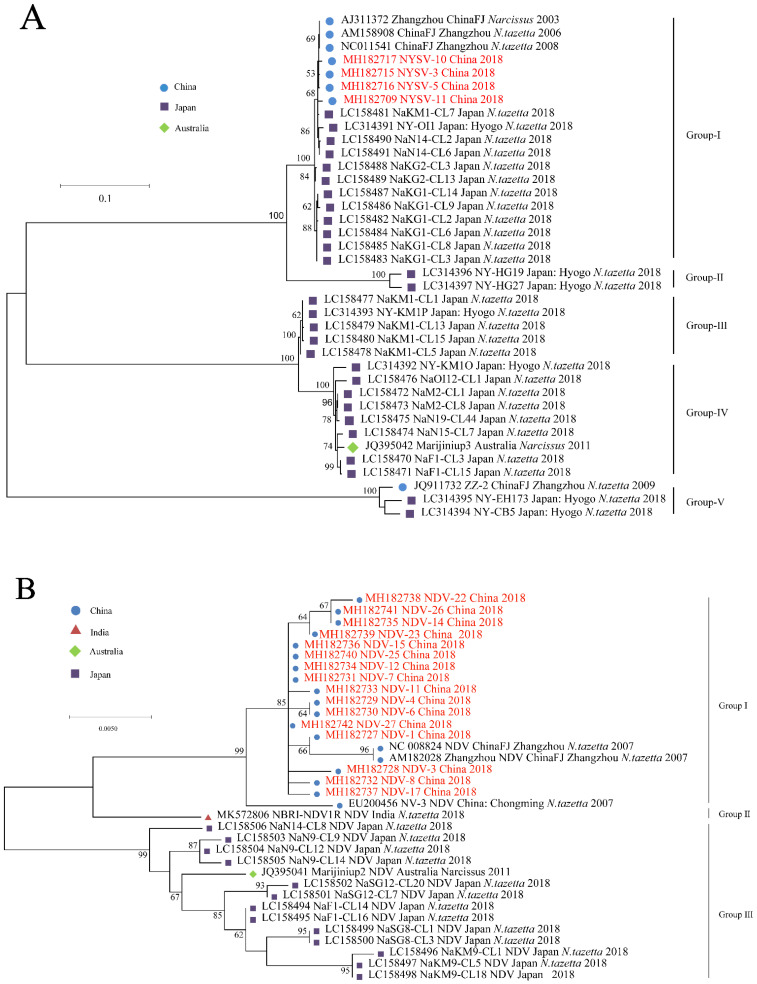

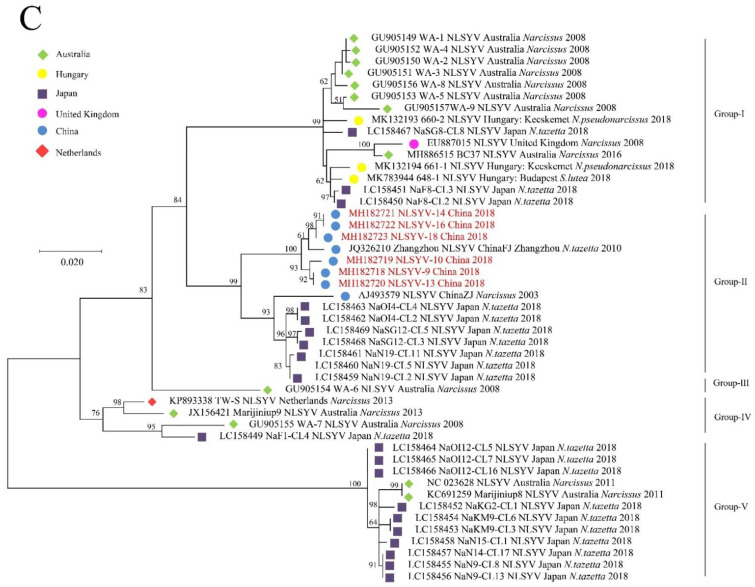
Maximum-likelihood tree was calculated from the coat protein gene sequences of the narcissus yellow stripe virus (**A**); narcissus degeneration virus (**B**) and narcissus late season yellows virus (**C**). Numbers at each node indicate the percentage of supporting bootstrap samples in maximum-likelihood trees. Isolates with red color are determined here.

Three recombinants were found in NYSV CP sequence data (Table S2) while no clear recombinants were found in the NDV and NLSYV CP sequences data. After the three NYSV recombinants were deleted, phylogenetic analyses were conducted based on alignments of the CP sequence, using the different methods mentioned above. NYSV, NDV and NLSYV are divided into five, three and five groups (Figure 2A–C), respectively, the grouping of isolates did correlate well with their geographical origins and isolates from Japan and China are often gathered in clusters (Figure 2). 

### 3.3. Nucleotide Composition Analysis

We analyzed the nucleotide composition of the CP coding sequences of the three narcissus viruses. The results revealed that the overall frequency of nucleotides (A%, C%, U% and G%) and GC content (GC%) of NYSV, NDV and NLSYV were (33.06 ± 0.47%, 20.60 ± 0.69%, 21.73 ± 0.83%, 24.60 ± 0.48% and 45.20 ± 1.10%), (33.54 ± 0.19%, 18.38 ± 0.21%, 24.57 ± 0.20%, 23.51 ± 0.18% and 41.89 ± 0.19%) and (33.72 ± 0.64%, 20.97 ± 0.52%, 20.72 ± 0.51%, 24.59 ± 0.38% and 45.55 ± 0.59%), respectively (Table S3). We observed that nucleotides A and U are the most abundant in the NDV CP coding sequences, while the NLSYV and NYSV are AG-rich. Further, the base contents in the third position of the three narcissus viruses (NYSV, NDV and NLSYV) were also calculated, and U3S%, C3S%, A3S% and G3S% in these viruses were (31.22% ± 0.023, 28.03% ± 0.031, 43.05% ± 0.032 and 26.20% ± 0.019), (39.59% ± 0.008, 20.92% ± 0.008, 41.07% ± 0.005 and 28.87% ± 0.006) and (28.99% ± 0.011, 27.74% ± 0.013, 44.04% ± 0.018 and 27.36% ± 0.013), respectively. From this, the most frequent nucleotide of all three viruses was A3S, followed by U3S. Additionally, the average GC contents of NYSV, NDV and NLSYV at the first, second and third positions (for GC12s and GC3s) of the CP coding sequences were (45.43 ± 0.24%, 41.80 ± 0.037%), (42.01 ± 0.18%, 37.01 ± 0.006%) and (45.36 ± 0.55%, 42.50 ± 0.012%), respectively. In addition, the content of the AU was also higher than that of the GC in CP sequences of the three viruses (Table S3). 

### 3.4. The RSCU Analysis

RSCU results show the frequency of gene codon usage of the three narcissus viruses (Table 1). In the NDV *CP* gene, there are 7 codons with RSCU values greater than 1.6, while there are 6 and 5 codons with RSCU values greater than 1.6 in the NLSYV and NYSV CP coding sequences and the highest RSCU values (2.78, 2.60 and 2.49), being those for CCA for the NDV, NLSYV and NYSV CP coding sequences, indicating extreme overrepresentation. Among the 18 preferred codons, no optional synonymous codons were underrepresented (RSCU < 0.6) for the three narcissus viruses CP coding sequences. The 14 preferred codons were U/A-ended (U-ended: 8; A-ended: 6) for NDV CP coding sequences (Table 1). A total of 14 and 12 of the 18 preferred codons were U/A- ended (U-ended: 5; A-ended: 9) (U-ended: 4; A-ended: 8) for the NLSYV and NYSV CP coding sequences (Table 1). This observation suggests that U- and A-ended codons were preferred in the three narcissus CP coding sequences. The above RSCU analysis results suggested that the preferred codons of the coding regions of the three narcissus viruses were affected by the restriction of nucleotide composition (A and U, in this case). In our RSCU analysis, to determine the potential influences of the narcissus on the codon usage patterns of the NLSYV, NYSV and NDV isolates, we calculated the RCSU value of narcissus genes. We found that the 18 preferred codons in the narcissus genes are all A/U ended, which means that there are consistent coding usage preferences between narcissus genes and the three narcissus CP coding sequences. Interestingly, CCA codons were similarly selected in the three viruses in CP coding regions and the narcissus genes, with values of 2.78, 2.60, 2.49 and 1.26, respectively.

### 3.5. Codon Usage Bias of the CP Coding Sequences

In the evaluation of gene codon usage bias based on ENC values, the mean ENC values of 55.78 ± 1.84%, 55.62 ± 0.74% and 54.79 ± 2.16% were described for NYSV, NDV and NLSYV CP coding sequences, respectively. In general, the smaller the ENC value, the higher the codon preference. It is also accepted that ENC values ≤35 are indicative of genes with a significant codon bias [32,33]. The ENC value indicated that codon usage bias is low in all three narcissus virus CP coding sequences.

### 3.6. Trends in Codon Usage Variations

**Figure 3:**
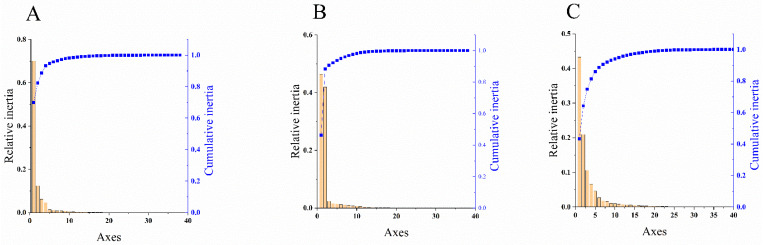
The relative and cumulative inertia of the 40 axes from a principal component analysis (PCA) of the relative synonymous codon usage (RSCU) values based on the narcissus yellow stripe virus (**A**); narcissus degeneration virus (**B**) and narcissus late season yellows virus (**C**) CP sequences. The relative (orange bars) and cumulative plot (blue squares) show 40 factors from a principal component.

**Figure 4:**
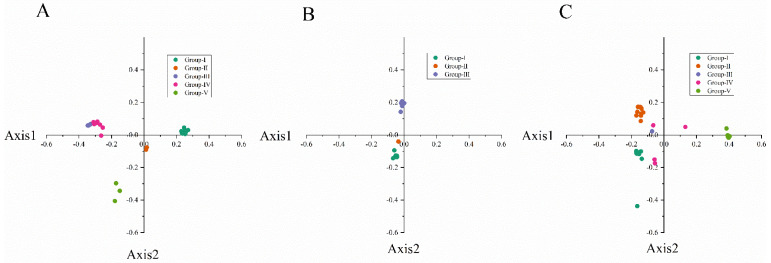
Correspondence analysis of synonymous codon usage towards the codons in narcissus yellow stripe virus (**A**); narcissus degeneration virus (**B**) and narcissus late season yellows virus (**C**) CP sequences. The analysis is based on the relative synonymous codon usage (RSCU) values of 59 synonymous codons. Different groups are represented by different colors. Among them, NYSV and NLSYV are divided into five groups (Group Ⅰ, Ⅱ, Ⅲ, Ⅳ, Ⅴ), which are represented by dark green, orange, purple, dark red and light green, respectively. NDV is divided into three groups (Group Ⅰ, Ⅱ, Ⅲ) with dark green, orange and purple, respectively.

PCA is used to analyze the fluctuation of codon RSCU value, which is a multivariate statistical method to explore the codon usage bias [42]. It can be seen from Figure 3 that the first principal component (PC1) of CP coding sequences of NYSV, NDV and NLSYV accounted for 93.28%, 92.32% and 81.41% of the total variation, respectively. The values of the first four axes for NYSV CP coding sequences were 70.01, 12.35, 6.25 and 4.67% (Figure 3A), while those observed for NDV and NLSYV CP were (46.37, 41.39, 2.41 and 1.61%), (43.30, 20.94, 10.62 and 6.55%), respectively (Figure 3B,C). These values showed that axis 1 was the dominant factor affecting codon usage for the *CP* gene. In this study, we accessed the PCA to determine the distribution of the CP coding region in different groups based on the RSCU values on the values of the first two axes. The PCA for the three viruses’ *CP* genes demonstrated few overlapped sites among the different groups, implying that the process of codon bias formation is not consistent (Figure 4).

### 3.7. The ENC-Plot Analysis

**Figure 5:**
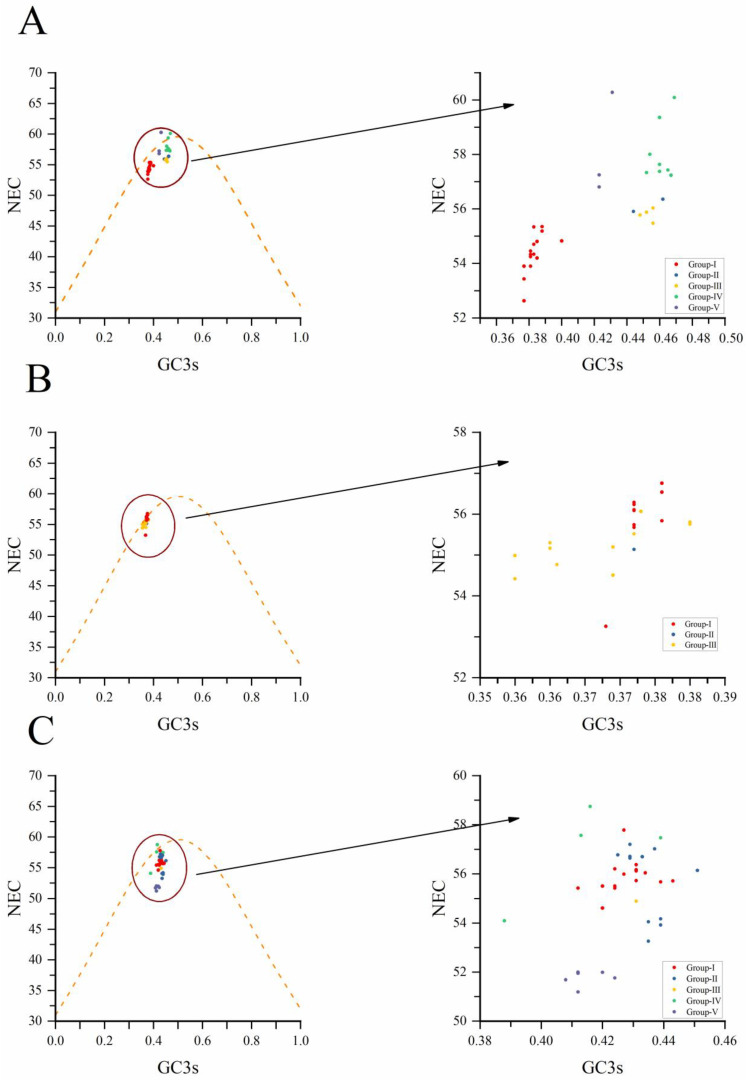
Effective Number of Codons (ENC) analysis of narcissus yellow stripe virus (**A**), narcissus de-generation virus (**B**) and narcissus late season yellows virus (**C**) coding sequences against GC3s. Effective Number of Codons (ENC) analysis of each coding sequence against GC3s. ENC is the number of effective codons. GC3s nucleotide is the frequency of G + C at the third position of the synonymous codon. The yellow curve (standard curve) shows the relationship between ENC values and GC3s under the random codon usage assumption. Different groups are marked with different colors. Among them, narcissus yellow stripe virus and narcissus late season yellows virus are divided into five groups (Group Ⅰ, Ⅱ, Ⅲ, Ⅳ, Ⅴ) which are represented by red, blue, yellow, green and purple, respectively. Narcissus degeneration virus is divided into three groups (Group Ⅰ, Ⅱ, Ⅲ), which are represented by red, blue, and yellow. The panel on the right graph corresponds to the left graph ABC’s enlarged detailed data graph.

We conducted an ENC-plot analysis for GC3s to study the factors influencing the codon usage bias of NYSV, NDV and NLSYV according to the CP coding sequences. It can be seen from Figure 5 that most of the *CP* genes of these three viruses are distributed under or near the standard curve. The correlation analysis between ENC and GC3s shows that the points fall below the standard curve. These imply that the codon preference is being affected by selection pressure rather than mutation pressure, whereas mutation pressure indicates when the data points up the standard curve [43]. In the CP coding sequence plots, NYSV, NDV and NLSYV isolate from different groups mostly clustered together below the standard ENC curve (Figure 5A–C), indicating that natural selection plays a greater role in the formation of NYSV, NDV and NLSYV *CP* gene preference.

### 3.8. Neutrality Plot

**Figure 6:**
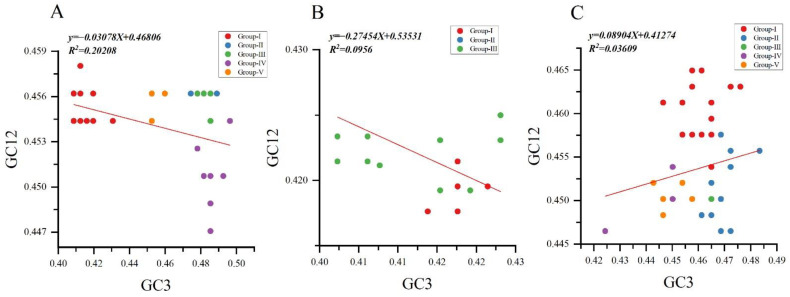
Neutrality plot analysis of the *CP* genes of narcissus yellow stripe virus (**A**); narcissus degeneration virus (**B**) and narcissus late season yellows virus (**C**) is shown. The abscissa and ordinate are GC3 and GC12, respectively. A value of 0.5 on the abscissa and ordinate indicates that the GC12 usage rate is equal to GC3. Different groups are marked with different colors.

To assess the degree of mutational pressure and natural selection on the codon usage in NYSV, NDV and NLSYV CP encoding sequence, we performed the neutrality analyses between GC12 and GC3 separately for the three viruses’ CP sequences. A negative correlation was observed between the GC12 and GC3 values for NYSV and NDV CP sequences. The slopes of linear regression were −0.03078, −0.2745 and 0.08904 for NYSV, NDV and NLSYV CP sequences, indicating mutation pressure accounted for 3.07%, 27.45% and 8.90% of the selection force, and natural selection accounted that for 96.93%, 72.55% and 91.1% for NYSV, NDV and NLSYV, respectively (Figure 6A–C). Thus, neutrality analysis has further shown that natural selection dominated the forces driving the CP codon usage bias of NYSV, NDV and NLSYV.

### 3.9. Parity Analysis

**Figure 7:**
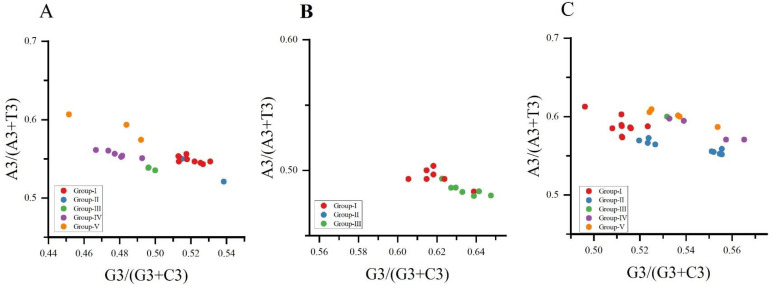
The AU (A3%/ (A3% + U3%)) and GC (G3%/ (G3% + C3%)) bias of the *CP* genes of narcissus yellow stripe virus (**A**); narcissus degeneration virus (**B**) and narcissus late season yellows virus (**C**) is shown. The center of the plot (both of the coordinates are 0.5) indicates a position where there is no bias. Group Ⅰ, Ⅱ, Ⅲ, Ⅳ and Ⅴ are represented by red, blue, green, purple and yellow dots, respectively.

We performed a PR2 bias plot analysis of the composition of the four bases on the third nucleotide of the three viruses’ codons. The results showed that the genes of the three viruses, NYSV, NDV and NLSYV (Figure 7A–C), are not evenly distributed in the plan. Normally, the center of the plot (the frequencies of A = U and G = C), where both coordinates are 0.5, indicates no bias is present in the selection or mutation forces [39]. It shows that the preference of the *CP* gene codon usage of the three viruses is not only affected by mutation pressure, but also may be affected by other factors such as selection pressure.

## 4. Discussion

In this paper, the codon usage pattern of NYSV, NDV and NLSYV *CP* gene and its main influencing factors were discussed and compared. Three *CP* genes of narcissus viruses were used for RSCU calculation and analysis to determine the use of high-frequency codons in narcissus viruses. The results showed that the codons generally preferred to use the third codon position of A/U. The results are consistent with the codon usage bias of banana bract mosaic virus (BBrMV), soybean mosaic virus (SMV) and citrus tristeza virus (CTV) [38,44,45]. It is worth noting that the best optimal codon of the three narcissus viruses is CCA, which may be related to the range of the codon usage bias difference and the genetic relationship of the species. The closer the genetic relationship is, the smaller the codon bias difference is, and the frequency of codon usage is often similar [38]. In summary, it is likely that the nucleotide compositions of potyviruses are strongly affected by the codon usage preferences of the viruses and the host plants, since potyviruses adopt the polyprotein-processing genome strategy, and the majority of the viral genome is the large open reading frame of the single gene of the polyprotein. The translation of this large open reading frame is entirely dependent on the tRNAs of the host plants [46,47].

The higher the ENC value is, the lower the codon bias is, and vice versa. In this paper, the average values of NYSV, NDV and NLSYV were 55.78, 55.62 and 54.79, respectively, indicating that codon bias was low. The low codon usage bias was also observed in the sugarcane mosaic virus (SCMV), potato virus M (PVM), broad bean wilt virus 2 (BBWV2) and papaya ringspot virus (PRSV) [48,49,50,51]. Studies have found that low codon usage bias reduces the competition for the synthesis mechanism between viruses and hosts, which is conducive to the expression of viral genes in host cells. We consider that the same three narcissus virus *CP* gene low codon bias is conducive to better adapting to its host [52,53].

The codon usage bias is the comprehensive result of the pressure mutation and natural selection of organisms in the evolution process. The optimal codon can improve the efficiency and accuracy of translation [54]. Considering that the codon usage pattern is affected by many factors in the formation process, the PCA is a multivariate statistical method to explore the codon usage bias. The PR2-plot, ENC-GC3s plot analysis and neutral plot analysis are used to evaluate the strength of the influence of natural selection and mutation pressure on codon usage preference. Natural selection is the main factor density affecting the codon usage bias. Some studies also found that natural selection played an important role in codon usage preferences of the potato virus X (PVX), rice stripe virus (RSV) and rice black-streaked dwarf virus (RBSDV) [55,56,57].

In summary, our results revealed the codon usage bias of narcissus viruses (NYSV, NDV and NLSYV), which can provide a certain scientific basis for the study of the molecular evolution of narcissus viruses.

## Figures and Tables

**Table 1:** The relative synonymous codon usage (RSCU) value of 59 codons encoding 18 amino acids according to the coat protein of three narcissus viruses and the RSCU value of narcissus genes.

Codon	aa	CP			Narcissus
		NDV	NLSYV	NYSV	
TTT	F	**1.46** *	**1.18**	**1.21**	**1.04**
TTC	F	0.54	0.82	0.79	0.96
TTA	L	**1.93**	0.70	1.08	1.23
TTG	L	1.40	**1.53**	**1.21**	1.27
CTT	L	1.00	1.18	0.88	**1.31**
CTC	L	0.35	0.56	1.14	0.87
CTA	L	0.66	0.70	1.10	0.64
CTG	L	0.67	1.33	0.59	0.68
ATT	I	**1.50**	0.77	**1.20**	**1.24**
ATC	I	0.86	**1.17**	0.78	0.97
ATA	I	0.64	1.06	1.01	0.79
GTT	V	1.09	1.32	1.03	**1.36**
GTC	V	1.09	0.62	0.55	0.74
GTA	V	0.24	0.43	0.97	0.94
GTG	V	**1.58**	**1.62**	**1.45**	0.95
TCT	S	0.90	0.04	0.63	**1.35**
TCC	S	0.42	0.32	0.11	0.98
TCA	S	1.31	**2.33**	**2.31**	1.09
TCG	S	0.80	0.39	0.31	0.79
AGT	S	**2.04**	1.57	1.59	1.00
AGC	S	0.52	1.35	1.06	0.79
CCT	P	0.17	0.56	0.54	1.23
CCC	P	0.65	0.32	0.43	0.83
CCA	P	**2.78**	**2.60**	**2.49**	**1.26**
CCG	P	0.40	0.52	0.54	0.61
ACT	T	0.59	0.40	0.75	**1.43**
ACC	T	0.43	0.61	1.10	0.95
ACA	T	1.35	**2.31**	**1.42**	1.01
ACG	T	**1.63**	0.69	0.72	0.62
GCT	A	0.91	0.77	0.91	**1.49**
GCC	A	0.64	0.99	1.13	0.80
GCA	A	**1.32**	**1.55**	**1.22**	1.13
GCG	A	1.13	0.69	0.74	0.58
TAT	Y	**1.32**	0.93	**1.20**	**1.24**
TAC	Y	0.68	**1.07**	0.80	0.75
CAT	H	0.81	**1.05**	0.61	**1.25**
CAC	H	**1.19**	0.95	**1.39**	0.72
CAA	Q	**1.80**	**1.45**	**1.16**	**1.23**
CAG	Q	0.20	0.55	0.84	0.77
AAT	N	**1.24**	**1.03**	0.93	**1.25**
AAC	N	0.76	0.97	**1.07**	0.74
AAA	K	0.97	**1.24**	0.99	**1.08**
AAG	K	**1.03**	0.76	**1.01**	0.88
GAT	D	**1.31**	**1.27**	**1.14**	**1.38**
GAC	D	0.69	0.73	0.86	0.62
GAA	E	**1.01**	**1.09**	**1.39**	**1.14**
GAG	E	0.99	0.91	0.61	0.86
TGT	C	**1.94**	**1.47**	0.00	**1.15**
TGC	C	0.06	0.53	**2.00**	0.76
CGT	R	1.48	0.11	1.01	1.02
CGC	R	0.15	0.85	0.72	0.54
CGA	R	0.77	**1.97**	0.99	1.00
CGG	R	0.57	0.58	0.35	0.55
AGA	R	**2.13**	1.36	**1.65**	**1.69**
AGG	R	0.91	1.14	1.28	1.21
GGT	G	**1.48**	0.82	0.95	1.06
GGC	G	0.67	**1.66**	0.48	0.64
GGA	G	1.25	1.17	**1.92**	**1.46**
GGG	G	0.61	0.34	0.66	0.84

* The most frequently used codons are shown in bold.

## Data Availability

All data presented in this study are available on request from the corresponding authors.

## Supplementary Materials

The following supporting information can be downloaded at: https://www.mdpi.com/article/10.3390/v14050846/s1, Table S1. The potyviruses of narcissus degeneration virus (NDV), narcissus late season yellows virus (NLSYV), and narcissus yellow stripe virus (NYSV) from the Chinese narcissus plants in Jiangsu province, China; Table S2. Recombination in narcissus yellow stripe virus was shown by RDP v4.0 software; Table S3. The nucleotide compositions of NYSV, NDV and NLSYV CP coding sequences.
