# Supplementary material for: Synonymous Codon Usage Analysis of Three Narcissus Potyviruses
Source: Viruses. 2022 Apr 19;14(5):846. doi: 10.3390/v14050846 (PMC9143068; doi:10.3390/v14050846)
Supplement: Supplementary file 1 [file viruses-14-00846-s001.zip › viruses-1623775-supplementary.pdf]

viruses MDPI

Supplementary Materials

Synonymous Codon Usage Analysis of Three Narcissus Potyviruses

Table S1. The three potyviruses detected in this study. The 26 CP gene sequences of the three narcissus viruses were sequenced by ourselves.

Virus	Isolate	Accession Number	Time
NDV	NDV-1	MH182727	2018
NDV	NDV-3	MH182728	2018
NDV	NDV-4	MH182729	2018
NDV	NDV-6	MH182730	2018
NDV	NDV-7	MH182731	2018
NDV	NDV-8	MH182732	2018
NDV	NDV-11	MH182733	2018
NDV	NDV-12	MH182734	2018
NDV	NDV-14	MH182735	2018
NDV	NDV-15	MH182736	2018
NDV	NDV-17	MH182737	2018
NDV	NDV-22	MH182738	2018
NDV	NDV-23	MH182739	2018
NDV	NDV-25	MH182740	2018
NDV	NDV-26	MH182741	2018
NDV	NDV-27	MH182742	2018
NLSYV	NLSYV-9	MH182718	2018
NLSYV	NLSYV-10	MH182719	2018
NLSYV	NLSYV-13	MH182720	2018
NLSYV	NLSYV-14	MH182721	2018
NLSYV	NLSYV-16	MH182722	2018
NLSYV	NLSYV-18	MH182723	2018
NYSV	NYSV-3	MH182715	2018
NYSV	NYSV-5	MH182716	2018
NYSV	NYSV-10	MH182717	2018
NYSV	NYSV-11	MH182709	2018

NDV, Narcissus degeneration virus; NLSYV, Narcissus late season yellows virus; NYSV, Narcissus yellow stripe virus.

Table S2. Recombination sites detected in the protein encoding regions of Narcissus yellow stripe virus.

**Isolate**	**Major parent**	**Minor parent**	**Recombination site**	**Recombination detecting program (*p*-value[a])**						
				RDP	GENECONV	BOOTSCAN	MAXCHI	CHIMAERA	SISCAN	3SEQ
JQ686724	UN[b]	MH182709	402-813	2.776×10^{-7}	7.052×10^{-18}	1.426×10^{-13}	7.265×10^{-17}	2.575×10^{-7}	3.445×10^{-12}	2.308×10^{-36}
KM066972	MH182709	LC314396	419-798	1.066×10^{-10}	5.050×10^{-3}	8.415×10^{-11}	7.798×10^{-44}	2.239×10^{-9}	1.295×10^{-4}	4.458×10^{-11}
KU516386	MH182709	LC314396	419-783	1.066×10^{-10}	5.050×10^{-3}	8.415×10^{-11}	7.798×10^{-44}	2.239×10^{-9}	1.295×10^{-4}	4.458×10^{-11}

[a]The analyses were done using default settings and a Bonferroni-corrected *p*-values cut-off of 0.01 in RDP4 software; [b]UN, Unknown
